# Prospective, randomized study comparing two different regimens of split-dose polyethylene glycol and their effect on endoscopic outcomes

**DOI:** 10.1186/s12876-024-03212-z

**Published:** 2024-04-12

**Authors:** Jawad Abou Zeid, Souheil Hallit, Bassem Akiki, Zeina Abou Zeid, Charbel Yazbeck

**Affiliations:** 1 School of Medicine and Medical Sciences, Holy Spirit University of Kaslik, Jounieh, P.O. Box 446, Lebanon; 2 Applied Science Research Center, Applied Science Private University, 11931 Amman, Jordan; 3 Department of Gastroenterology, Notre-Dame des Secours University Hospital, Byblos, Lebanon

**Keywords:** Polyethylene glycol, Split dose, Gastric fullness, Colon cancer, Adequate bowel preparation, Boston bowel preparation score, Adenoma detection rate

## Abstract

**Background:**

Different split regimens of polyethylene glycol are routinely used and no guidelines are available to select an optimal protocol of ingestion. This study aims to compare the efficacy and side effect profile of two different regimens of polyethylene glycol bowel preparation solution: PEG (3 + 1) vs. PEG (2 + 2).

**Methods:**

240 patients above the age of 18 years were included in the study between June 1st and November 31^st,^ 2023. Patients were randomly assigned either to Group A, consisting of 115 patients receiving a 3 L of PEG the night before the colonoscopy, and 1 L the same morning of the procedure. Or to group B, where 125 patients ingested 2 L the night before the procedure, and the remaining 2 L the same morning. The cleansing efficacy was evaluated by the attending endoscopist using the Boston Bowel Preparation Scale, through a score assigned for each segment of the colon (0–3). Side effects, tolerability, and willingness to retake the same preparation were listed by an independent investigator using a questionnaire administered before the procedure.

**Results:**

A higher percentage of patients had gastric fullness with the 3 + 1 vs. 2 + 2 preparation (58.3% vs. 31.2%; *p* <.001). A higher Boston bowel preparation score was seen in patients who took the 2 + 2 vs. 3 + 1 preparation (7.87 vs. 7.23). Using the 2 + 2 preparation was significantly associated with higher Boston bowel preparation scores vs. the 3 + 1 preparation (OR = 1.37, *p* =.001, 95% CI 1.14, 1.64). After adjustment over other variables (age, gender, comorbidities, previous abdominal surgeries, presence of adenoma, and time between last dose and colonoscopy), results remained the same (aOR = 1.34, *p* =.003, 95% CI 1.10, 1.62).

**Conclusion:**

While both (2 + 2) and (3 + 1) regimens of polyethylene glycol are a good choice for a successful colonoscopy, we recommend the use of (2 + 2) regimen for its superior efficacy in bowel cleansing.

## Background

For years, colonoscopy has become a routinely performed procedure in adult people; it is the gold standard procedure for the evaluation of colonic abnormalities, as well as for surveillance and screening for the presence of a colorectal malignancy [1]. It is well known that the success of the procedure and a good intestinal mucosal visualization correlate directly with the quality of the pre-endoscopic bowel preparation [2]. An inadequately prepared bowel may lead to failure or prolonged time of the procedure (decreased cecal intubation rate, decreased adenoma detection rate, missed colonic polyps) while increasing the likelihood of complications [3].

An optimal bowel cleansing before colonoscopy remains one of the main obstacles we face during the procedure [4, 5]. Polyethylene glycol (PEG) and sodium picosulfate/magnesium citrate (SPMC) stand among the most commonly used bowel preparations before colonoscopy.

A main advantage of SPMC is the relatively lower volume of preparation that the patient has to ingest prior to colonoscopy [1].. This is believed to happen due to the reduced effective volume of the SPMC preparation 2 L compared to the 4 L PEG solution [1, 6]. However, many contraindications limit the use of SPMC, and many adverse side effects compromise its use in patients willing to undergo colonoscopy [1, 7, 8].

As opposed to SPMC preparation, PEG is a balanced electrolyte lavage rather than an osmotic agent, also known as an isosmotic nonabsorbable polymer. What makes it distinct from other bowel preparations is its relatively minimal impact on serum electrolytes and intravascular blood volume. Furthermore, it offers the possibility to perform colonoscopies on patients within 12 to 18 h of the initial evaluation with minimal diet restriction [2]. Besides, the high-volume PEG bowel preparation remains safe even in patients with comorbidities such as hepatic dysfunction, acute or chronic kidney disease, heart disease, or electrolyte imbalance [12].

On the other hand, different centers of colonoscopy worldwide have used distinct protocols for the administration of PEG. Among the most widely used PEG protocols we mention, whole non-split dose 4 L of PEG, split dose (3 L + 1 L), and split dose (2 L + 2 L). Analyzing the studies previously published on the subject, we can find several randomized trials and meta-analysis revealing that split-dose PEG offers major benefits in clinical practice and more satisfactory scores of bowel cleansing efficacy when compared to a whole non-split dose of PEG [13]. More importantly, patient willingness to retake the same was improved when splitting the dose, along with a decreased frequency of nausea as stated in a meta-analysis [14–16].

In addition, previous studies consider the timing of PEG ingestion as an important factor influencing the effectiveness of bowel preparation [13]. Likewise, the European Society of gastrointestinal endoscopy recommends that the interval of time between the ingestion of the last dose of the preparation and the procedure itself should be decreased and not exceed 4 h [6, 17, 18]. The idea behind having a certain dose of PEG ingested as close as possible to colonoscopy is attributed to the fluid, bile, and debris that accumulate overnight and after the first dose of PEG has been fully taken. Hence, this will result in poor mucosal visualization during endoscopy. What also became standard is that the American society of Anesthesiologists recommend that patients should have nothing taken by mouth at least 2 h prior to the procedure in order to minimize the pulmonary aspiration risk [17].

Searching the literature, we could not find data that supports the use of a standardized, statistically proven, effective way to split the 4 L of PEG. For this reason, we compared the patient compliance, tolerability, willingness to retake the preparation, and cleansing efficacy of the 2 most used protocols of split dose PEG aiming to find an optimal regimen. The first protocol tested consists of a 4 L (3 L + 1 L) split dose, with 3 L ingested the night prior to the procedure and the remaining 1 L the same morning of the procedure. The second protocol is a 4 L (2 L + 2 L) split dose, among which 2 L ingested the night prior to the procedure and 2 L the same morning of the procedure.

## Methods

### Patients/Participants

**Fig. 1:**
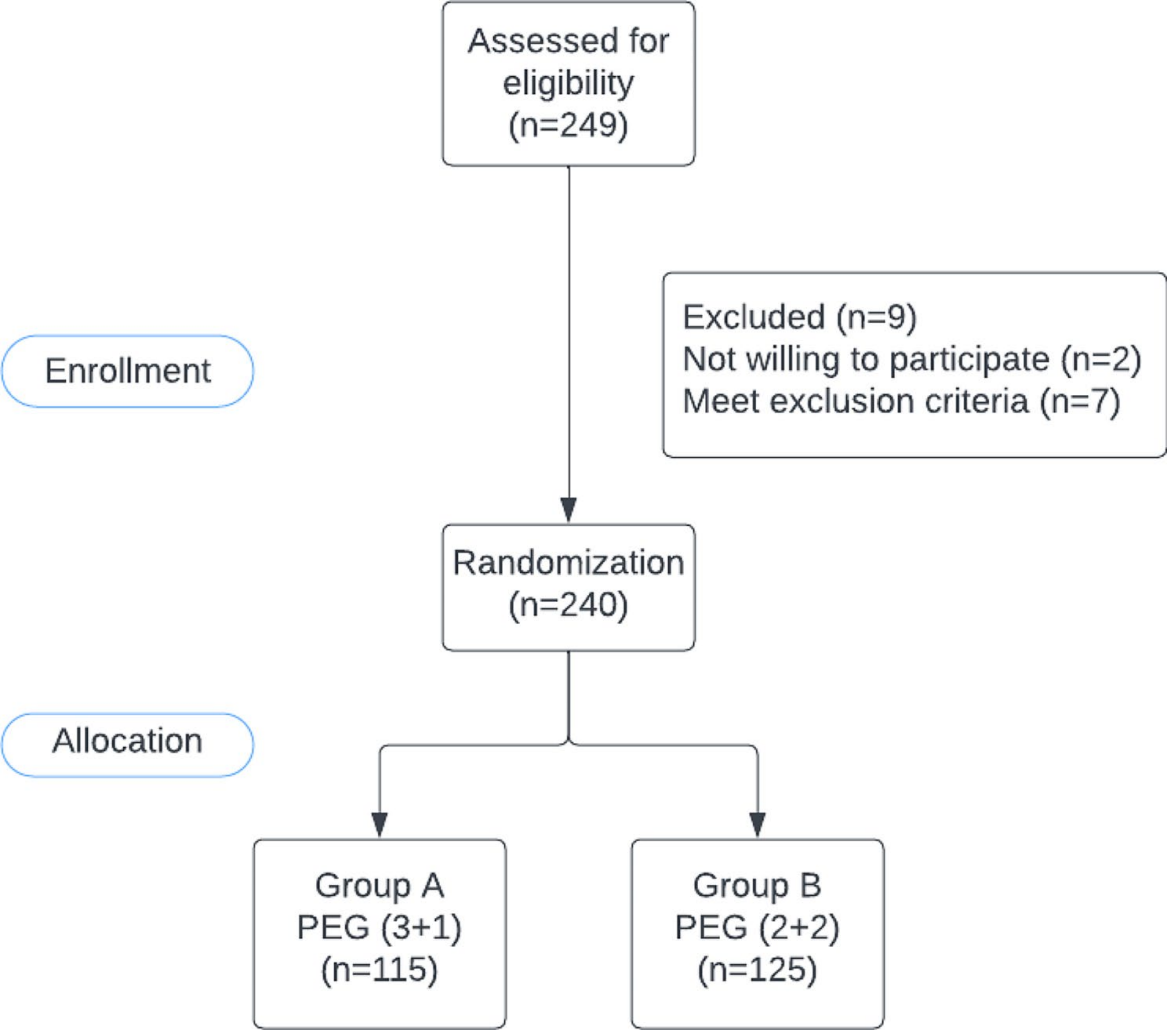
Flow chart for the patients selection in the study

Patients included in the study are those who presented to Notre Dame de Secours University Hospital for elective colonoscopy. The study was performed at Notre Dame de Secours between June 1, 2023, and November 31, 2023; 240 patients were enrolled (Fig. 1). After informed consent, the patients were randomly provided written instructions on either of the two PEG bowel preparations by their respective endoscopist (a computer-generated randomization sheet was used by the attending physicians to allocate each colonoscopy candidate either to group A or group B). Exclusion criteria were as follows: Patients under the age of 18 years; the presence of serious medical conditions, such as severe cardiac or renal disease, active alcoholism, drug addiction, metabolic disease, or major cognitive/psychiatric illness; previous allergy to polyethylene glycol or refusal of consent to participate in the study. Colonoscopies were performed by one of three staff endoscopists. All procedures were completed between 8:00 AM and 1 PM under sedation using propofol I.V with continuous monitoring of heart rate and oxygen saturation by the anesthesia team during the procedure and recovery time.

### Minimum sample size

The G-power software calculated a minimum sample of 191 patients based on a hypothetical small-to-moderate effect size of 0.2 between the type of regimen and the Boston Bowel Preparation Scale (BBPS) score (in the absence of similar studies tackling the same objective), an alpha error of 5% and a power of 80%.

### Preparation instructions

Patients were randomly assigned to receive one of two preparation regimens. **Group A** were to ingest a split dose of PEG by using 4 sachets (Fortrans®; Beaufour, France), each containing 64 g macrogol 4000, 5.7 g anhydrous sodium sulfate, 1.68 g sodium bicarbonate, 1.46 g sodium chloride, and 0.75 g potassium chloride. These 4 sachets are to be dissolved in 4 L water, 3 L of the solution ingested between 5:00 PM and 9:00 PM the evening prior to the procedure, and the remaining 1 L the same morning of to the procedure at least 2 h prior to the colonoscopy in order to minimize aspiration risk after sedation. Patients in **Group B** were instructed to ingest 2 L the evening prior to the procedure and 2 L the same morning of the procedure. In both groups, patients were instructed to adhere to a low residue diet the day before colonoscopy followed by a liquid only diet starting at 4 pm. Investigators were not blinded to the preparation type, as the attending physician had to give detailed instructions for the patients and ensure a good understanding of the assigned ingestion protocol.

### Data collection

An independent investigator using the ‘Mayo clinic bowel preparation and tolerability questionnaire’ interviewed each candidate before colonoscopy [19]. This is a validated questionnaire. It consists of nine items: evaluation of overall tolerability and willingness to retake the same preparation, the baseline number of bowel movements, any potential health issues that might have interfered with the bowel preparation, add to that the presence of common symptoms experienced during bowel preparation (nausea, vomiting bloating, gastric fullness, bad taste in mouth, and lack of sleep from excessive bathroom trips). Then in terms of compliance patients were asked about the amount of remaining solution after completing the drink; patients who did not complete their preparation as instructed were asked to estimate the total residual amount of PEG to the closest 100 mL.

As a secondary outcome, the efficacy of bowel preparation and the quality of mucosa visualization were evaluated by one of the 3 endoscopists staff using the BBPS [20] by scoring each of the 3 segments of the colon: right-sided colon, transverse colon, and left-sided colon. Each segment can be scored between 0 and 3, so the total score ranges from 0 to 9. In addition, we evaluated: (a) the adenoma detection rate (ADR) each time an adenoma was confirmed it was noted in the patient file, and (b) adequate bowel preparation defined as a global BPPS score ≥ 6. (c) cecal intubation rate, defined as the percentage of colonoscopies reaching and visualizing the whole cecum. (d) ileal intubation, defined as whether the endoscopist has reached the terminal ileum with the scope.

### Statistical analysis

The SPSS software v.26 was used for the statistical analysis. The Chi-2 test was used to compare two categorical variables and the Student t test to compare two means. Logistic regressions were used to calculate the unadjusted/adjusted odds ratios before and after adjustment over variables respectively. *P* <.05 was deemed statistically significant.

## Results

A significantly lower mean age was seen in patients who took the 2 + 2 vs. 3 + 1 preparation (51.90 vs. 56.05; *p* =.027) (Table 1). A higher percentage of patients had gastric fullness with the 3 + 1 vs. 2 + 2 preparation (58.3% vs. 31.2%; *p* <.001) (Table 2). Finally, a higher Boston bowel preparation score was seen in patients who took the 2 + 2 vs. 3 + 1 preparation (7.87 vs. 7.23) (Table 3).

Note that for the following tables, numbers in bold indicate significant p values. Group 3 + 1 refers to patients who took 3 L of the preparation the night before the colonoscopy and 1 L the day of the procedure. Group 2 + 2 refers to patients who took 2 L of the preparation the night before the colonoscopy and 2 L the day of the procedure.

**Table 1:** Patients demographics and characteristics

	3 + 1 (*n* = 115)	2 + 2 (*n* = 125)	Total (*n* = 240)	*p*
Gender				0.646
Male	61 (53.0%)	70 (56.0%)	131 (54.6%)	
Female	54 (47.0%)	55 (44.0%)	109 (45.4%)	
Age (years)	56.05 ± 14.56	51.90 ± 14.39	53.89 ± 14.59	**0.027**
Comorbidities				0.282
No	55 (48.2%)	69 (55.2%)	124 (51.9%)	
Yes	59 (51.8%)	56 (44.8%)	115 (48.1%)	
Previous abdominal surgery				0.242
No	80 (69.6%)	78 (62.4%)	158 (65.8%)	
Yes	35 (30.4%)	47 (37.6%)	82 (34.2%)	

Numbers in bold indicate significant *p* values

**Table 2:** Questionnaire evaluating side effects, tolerability and willingness to retake the preparation

	3 + 1 (*n* = 115)	2 + 2 (*n* = 125)	Total (*n* = 240)	*p*
Bad taste in the mouth				0.511
No	59 (51.8%)	70 (56.0%)	129 (54.0%)	
Yes	55 (48.2%)	55 (44.0%)	110 (46.0%)	
Gastric fullness				**< 0.001**
No	48 (41.7%)	86 (68.8%)	134 (55.8%)	
Yes	67 (58.3%)	39 (31.2%)	106 (44.2%)	
Lack of sleep				0.874
No	65 (57.0%)	70 (56.0%)	135 (56.5%)	
Yes	49 (43.0%)	55 (44.0%)	104 (43.5%)	
Nausea/Vomiting				0.518
No	68 (59.1%)	79 (63.2%)	147 (61.3%)	
Yes	47 (40.9%)	46 (36.8%)	93 (38.8%)	
Bloating/gas				0.629
No	72 (62.6%)	82 (65.6%)	154 (64.2%)	
Yes	43 (37.4%)	43 (34.4%)	86 (35.8%)	
Abdominal pain				0.774
No	91 (79.1%)	97 (77.6%)	188 (78.3%)	
Yes	24 (20.9%)	28 (22.4%)	52 (21.7%)	
Willing to drink the same preparation again				0.741
No	20 (17.5%)	24 (19.2%)	44 (18.4%)	
Yes	94 (82.5%)	101 (80.8%)	195 (81.6%)	
Amount of bowel preparation left in the bottle after drinking it to your best effort	0.16 ± 0.33	0.11 ± 0.57	0.12 ± 0.35	0.413
Tolerability of the bowel preparation	3.47 ± 1.14	3.36 ± 1.29	3.42 ± 1.22	0.488

Numbers in bold indicate significant *p* values

**Table 3:** Data concerning the indicators of cleansing efficacy

	3 + 1 (*n* = 115)	2 + 2 (*n* = 125)	Total (*n* = 240)	p
Boston bowel preparation score	7.23 ± 1.61	7.87 ± 1.28	7.56 ± 1.48	**0.001**
Presence of adenoma				0.360
No	87 (75.7%)	88 (70.4%)	175 (72.9%)	
Yes	28 (24.3%)	37 (29.6%)	65 (27.1%)	
Ileal intubation				0.612
No	14 (12.2%)	18 (14.4%)	32 (13.3%)	
Yes	101 (87.8%)	107 (85.6%)	208 (86.7%)	

Numbers in bold indicate significant *p* values

### Multivariable analysis

Using the 2 + 2 preparation was significantly associated with higher Boston bowel preparation scores vs. the 3 + 1 preparation (OR = 1.37, *p* =.001, 95% CI 1.14, 1.64). After adjustment over other variables (age, gender, comorbidities, previous abdominal surgeries, presence of adenoma, and time between last dose and colonoscopy), results remained the same (aOR = 1.34, *p* =.003, 95% CI 1.10, 1.62).

## Discussion

For many years, PEG solution has been the mainstay for preparing patients for colonoscopy [10]. Ingestion of a whole non-split dose of PEG solution is reasonable in the only case where the procedure is to be performed in the afternoon. Otherwise, a split dose of PEG is the rule. In our present study, we are comparing the group A/ (3 + 1) regimen to the group B/ (2 + 2) regimen. Our study findings unveiled a statistically significant improvement in bowel cleansing using the (2 + 2) preparation compared to (3 + 1) with a BBPS of 7.81 vs. 7.23 respectively. This enhanced mucosal visualization in group B is most probably correlated to the two-liter volume that patients were to ingest the same morning of the procedure. We have previously mentioned that guidelines recommend the second dose of the solution to be as close as possible to the procedure [6, 17, 18], but not closer than 2 h in order to decrease the aspiration risk [21]. Now with the discussed results, we are additionally able to suggest that the second dose of the solution not only has to be close to the procedure, but also the volume has to be large enough to have a perfect cleansing.

Besides, the tolerability and large volume of the solution along with the associated adverse events are of major debate. Many researchers have found that patient compliance, tolerability, and willingness to retake the same preparation are positively affected each time the volume of the solution is smaller [9, 14, 22, 23]. Therefore, when comparing two high-volume solutions no significant difference is expected concerning the aforementioned factors. In our analysis, the difference between the 2 groups in terms of willingness to retake the preparation, patient tolerability, and compliance with the preparation, did not appear to be statistically significant. These similarities were predictable, as both groups received a same total of 4 L of a same ingredient solution. On top of that, PEG has a ‘really bad taste’ as described by the patients and they have a hard time drinking that huge amount in a short period. Then this makes sense when neither of the groups will be different in terms of the factors mentioned above. Although no significant difference was noted in adverse events (nausea, vomiting, abdominal pain, bloating, lack of sleep, bad taste) it is noteworthy to know that a significant difference exists in terms of gastric fullness. This is occurring more often in patients allocated to group A most likely because of the 3 L volume that these patients have to ingest in a very short time, leaving them with that annoying sensation of gastric fullness.

On the other hand, the adenoma detection rate is a measure of superiority for colonoscopy and is a primary indicator of the quality of mucosal visualization [24]. The ADR is perversely linked with colorectal cancer. Furthermore, Cecal intubation is considered a quality indicator as it decreases the cost by eliminating the need to repeat the procedure to complete the colonoscopy [24, 25]. Better bowel preparation is associated with higher cecal intubation rates and higher adenoma detection rates [26]. Cecal intubation is necessary since it has been consistently shown that a significant portion of colorectal neoplasms are situated in the proximal colon and it is now being known that poor bowel preparation results in lower cecal intubation and it is recommended to have a rate of cecal intubation ≥ 90% [22]. Ileal intubation although it is not required in most colonoscopies, it remains the gold standard evidence for complete colonoscopy. Apart from that, an adequate bowel preparation is defined as a BPPS score ≥ 6 and it is suggested to have that target score in at least 90% of the cases based on previous randomized clinical trials of split-dose bowel cleansing [27–29].

About ADR, cecal intubation, ileal intubation, and adequate bowel preparation, no pronounced dissimilarity is observed between the two groups compared. However, this similarity can be an indicator of good cleansing efficacy being achieved with both preparations, even though (3 + 1) regimen is inferior in terms of bowel cleansing, yet it achieves an efficacy congruent to that described in the literature. Our findings showed an adenoma detection rate of 27% in the total patients included in the study, a number superior to the 25%, the latter being described as the minimum standard in the European guidelines [30]. In addition, cecal intubation is strikingly achieved in 100% of the patients significantly higher than the recommended rate of 90%, this is undoubtedly attributed to the skills of the gastroenterologist in part and the good cleansing from another part. Parallel to that ileal intubation was achieved in 86.7% way above the 40.7% found in a large study realized by alkhatib et al. in 2022 [31]. Furthermore, adequate bowel preparation was achieved in 88.8% of the patients included in the study. This result is slightly inferior to the recommended target score, but is, however, equal or superior to other scores found in the literature.

### Limitations

First, in our study, we have compared two split dose regimens of PEG. Other possible combinations of PEG-based solutions are being used, and studies assessing the use of PEG with an add-on laxative were beyond our purview. Second, the assessment of the level of cleansing might be hampered by a degree of interoperator variability that we did not take into consideration. Third, we cannot eliminate a potential information bias, especially during patient interrogation. Finally, in our study, there is a chance of sub-optimal reproducibility with a single-center observational strategy. However, our results are supported by the large sample size and the prospective design of our study. Furthermore, additional advantages of our research consist in the use of validated scales including BPPS and mayoclinic patient questionnaire.

## Conclusion

The results of this study show that bowel preparation with the PEG-4 L split-dose (2 + 2) solution achieves significantly greater quality of bowel cleansing, with a markedly less sensation of gastric fullness and no added burden or discomfort to the patient compared to the (3 + 1) regimen of PEG. Further studies are required to look for a possible low-volume regimen of PEG (alone or in combination with other laxatives) that can furtherly decrease the burden of the preparation on the patient while maintaining or even improving the quality of bowel cleansing.

## Data Availability

All data generated or analyzed during this study are not publicly available due to restrictions from the ethics committee, but are available upon a reasonable request from the corresponding author (SH).

## References

[1] de Miranda Neto AA, de Moura DTH, Hathorn KE, Tustumi F, de Moura EGH, Ribeiro IB. Efficacy and Patient Tolerability of Split-Dose Sodium Picosulfate/Magnesium Citrate (SPMC) Oral Solution Compared to the Polyethylene Glycol (PEG) Solution for Bowel Preparation in Outpatient Colonoscopy: An Evidence-Based Review, *Clin Exp Gastroenterol*, vol. 13, pp. 449–457, Oct. 2020, 10.2147/CEG.S237649. 10.2147/CEG.S237649 PMC7548852 33116741

[2] Ben Chaabane N, et al. Préparation intestinale avant coloscopie. La Presse Médicale. Jan. 2012;41(1):37–42. 10.1016/j.lpm.2011.04.017. 10.1016/j.lpm.2011.04.017 21795010

[3] Hassan C et al. Aug., Bowel preparation for colonoscopy: European Society of Gastrointestinal Endoscopy (ESGE) Guideline - Update 2019, *Endoscopy*, vol. 51, no. 8, pp. 775–794, 2019, 10.1055/a-0959-0505. 10.1055/a-0959-0505 31295746

[4] Shahini E, et al. Factors affecting the quality of bowel preparation for colonoscopy in hard-to-prepare patients: evidence from the literature. World J Gastroenterol. Mar. 2023;29(11):1685–707. 10.3748/wjg.v29.i11.1685. 10.3748/wjg.v29.i11.1685 PMC10107216 37077514

[5] Wexner SD et al. Jun., A consensus document on bowel preparation before colonoscopy: prepared by a task force from the American Society of Colon and Rectal Surgeons (ASCRS), the American Society for Gastrointestinal Endoscopy (ASGE), and the Society of American Gastrointestinal and Endoscopic Surgeons (SAGES), *Gastrointest Endosc*, vol. 63, no. 7, pp. 894–909, 2006, 10.1016/j.gie.2006.03.918. 10.1016/j.gie.2006.03.918 16733101

[6] Kim YS, et al. Randomized clinical trial comparing reduced-volume oral picosulfate and a prepackaged low-residue diet with 4-liter PEG solution for bowel preparation. Dis Colon Rectum. Apr. 2014;57(4):522–8. 10.1097/DCR.0000000000000066. 10.1097/DCR.0000000000000066 24608310

[7] Lawrance IC, Willert RP, Murray K. Bowel cleansing for colonoscopy: prospective randomized assessment of efficacy and of induced mucosal abnormality with three preparation agents. Endoscopy. May 2011;43(5):412–8. 10.1055/s-0030-1256193. 10.1055/s-0030-1256193 21547879

[8] Kim HG et al. Jul., Sodium Picosulfate with Magnesium Citrate (SPMC) Plus Laxative Is a Good Alternative to Conventional Large Volume Polyethylene Glycol in Bowel Preparation: A Multicenter Randomized Single-Blinded Trial, *Gut Liver*, vol. 9, no. 4, pp. 494–501, 2015, 10.5009/gnl14010. 10.5009/gnl14010 PMC4477993 25287163

[9] Abdul-Baki H (2008). A randomized, controlled, double-blind trial of the adjunct use of tegaserod in whole-dose or split-dose polyethylene glycol electrolyte solution for colonoscopy preparation. Gastrointest Endosc.

[10] El AMA, Sayed et al. Jul., A randomized single-blind trial of whole versus split-dose polyethylene glycol-electrolyte solution for colonoscopy preparation, *Gastrointest Endosc*, vol. 58, no. 1, pp. 36–40, 2003, 10.1067/mge.2003.318. 10.1067/mge.2003.318 12838218

[12] Pontone S, et al. Polyethylene glycol-based bowel preparation before colonoscopy for selected inpatients: a pilot study. J Dig Dis. Jan. 2018;19(1):40–7. 10.1111/1751-2980.12571. 10.1111/1751-2980.12571 29266839

[13] Park JS et al. Jul., Quality and effect of single dose versus split dose of polyethylene glycol bowel preparation for early-morning colonoscopy, *Endoscopy*, vol. 39, no. 7, pp. 616–619, 2007, 10.1055/s-2007-966434. 10.1055/s-2007-966434 17611916

[14] Pan H et al. Aug., Same-day single-dose vs large-volume split-dose regimens of polyethylene glycol for bowel preparation: A systematic review and meta-analysis, *World J Clin Cases*, vol. 10, no. 22, pp. 7844–7858, 2022, 10.12998/wjcc.v10.i22.7844. 10.12998/wjcc.v10.i22.7844 PMC9372824 36158495

[15] Enestvedt BK, Tofani C, Laine LA, Tierney A, Fennerty MB. 4-Liter split-dose polyethylene glycol is superior to other bowel preparations, based on systematic review and meta-analysis, *Clin Gastroenterol Hepatol*, vol. 10, no. 11, pp. 1225–1231, Nov. 2012, 10.1016/j.cgh.2012.08.029. 10.1016/j.cgh.2012.08.029 22940741

[16] Kilgore TW, et al. Bowel preparation with split-dose polyethylene glycol before colonoscopy: a meta-analysis of randomized controlled trials. Gastrointest Endosc. Jun. 2011;73(6):1240–5. 10.1016/j.gie.2011.02.007. 10.1016/j.gie.2011.02.007 21628016

[17] Church JM. Effectiveness of polyethylene glycol antegrade gut lavage bowel preparation for colonoscopy–timing is the key! *Dis Colon Rectum*, vol. 41, no. 10, pp. 1223–1225, Oct. 1998, 10.1007/BF02258217. 10.1007/BF02258217 9788383

[18] Froehlich F, Wietlisbach V, Gonvers J-J, Burnand B, Vader J-P. Impact of colonic cleansing on quality and diagnostic yield of colonoscopy: the European Panel of Appropriateness of Gastrointestinal Endoscopy European multicenter study, *Gastrointest Endosc*, vol. 61, no. 3, pp. 378–384, Mar. 2005, 10.1016/s0016-5107(04)02776-2. 10.1016/s0016-5107(04)02776-2 15758907

[19] Patel M, Staggs E, Thomas CS, Lukens F, Wallace M, Almansa C. Development and validation of the Mayo Clinic Bowel Prep Tolerability Questionnaire, *Digestive and Liver Disease*, vol. 46, no. 9, pp. 808–812, Sep. 2014, 10.1016/j.dld.2014.05.020. 10.1016/j.dld.2014.05.020 24953203

[20] Lai EJ, Calderwood AH, Doros G, Fix OK, Jacobson BC. The Boston bowel preparation scale: a valid and reliable instrument for colonoscopy-oriented research, *Gastrointest Endosc*, vol. 69, no. 3 Pt 2, pp. 620–625, Mar. 2009, 10.1016/j.gie.2008.05.057. 10.1016/j.gie.2008.05.057 PMC2763922 19136102

[21] Practice Guidelines for Preoperative Fasting and the Use of Pharmacologic Agents to Reduce the Risk of Pulmonary Aspiration: Application to Healthy Patients Undergoing Elective Procedures: An Updated Report by the American Society of Anesthesiologists Task Force on Preoperative Fasting and the Use of Pharmacologic Agents to Reduce the Risk of Pulmonary Aspiration, Anesthesiology, vol. 126, no. 3, pp. 376–393. Mar. 2017, 10.1097/ALN.0000000000001452. 10.1097/ALN.0000000000001452 28045707

[22] Chen J, Athilingam P, Brady P (2018). Does 2-L Split-Dose Polyethylene Glycol Bowel Preparation improve the quality of Screening Colonoscopy?. Gastroenterol Nurs.

[23] Jin Z, Lu Y, Zhou Y, Gong B. Systematic review and meta-analysis: sodium picosulfate/magnesium citrate vs. polyethylene glycol for colonoscopy preparation. Eur J Clin Pharmacol. May 2016;72(5):523–32. 10.1007/s00228-016-2013-5. 10.1007/s00228-016-2013-5 26818765

[24] Rex DK, et al. Quality indicators for colonoscopy. Gastrointest Endosc. Jan. 2015;81(1):31–53. 10.1016/j.gie.2014.07.058. 10.1016/j.gie.2014.07.058 25480100

[25] Yan H et al. Dec., 3 L split-dose polyethylene glycol is superior to 2 L polyethylene glycol in colonoscopic bowel preparation in relatively high-BMI (≥ 24 kg/m2) individuals: a multicenter randomized controlled trial, *BMC Gastroenterol*, vol. 23, no. 1, p. 427, 2023, 10.1186/s12876-023-03068-9. 10.1186/s12876-023-03068-9 PMC10698874 38053082

[26] Mohamed R, Hilsden RJ, Dube C, Rostom A (2016). Split-Dose Polyethylene glycol is Superior to single dose for Colonoscopy Preparation: results of a Randomized Controlled Trial. Can J Gastroenterol Hepatol.

[27] Silva APD (2011). The prone 12 o’clock position reduces ileal intubation time during colonoscopy compared to the left lateral 6 o’clock (standard) position. BMC Gastroenterol.

[28] Börsch G, Schmidt G. Endoscopy of the terminal ileum. Diagnostic yield in 400 consecutive examinations, *Dis Colon Rectum*, vol. 28, no. 7, pp. 499–501, Jul. 1985, 10.1007/BF02554095. 10.1007/BF02554095 4017810

[29] Tang S-J, Wu R. Ilececum: A Comprehensive Review, *Can J Gastroenterol Hepatol*, vol. 2019, p. 1451835, 2019, 10.1155/2019/1451835. 10.1155/2019/1451835 PMC6378086 30854348

[30] Kaminski MF et al. Apr., Performance measures for lower gastrointestinal endoscopy: a European Society of Gastrointestinal Endoscopy (ESGE) Quality Improvement Initiative, *Endoscopy*, vol. 49, no. 4, pp. 378–397, 2017, 10.1055/s-0043-103411. 10.1055/s-0043-103411 28268235

[31] Alkhatib AA, Kumar S. Clinical yield of Ileal Intubation during Screening Colonoscopy. Cureus, 14, 1, p. e20870, 10.7759/cureus.20870. 10.7759/cureus.20870 PMC8803374 35145777

